# A new long-term measure of sustainable growth under uncertainty

**DOI:** 10.1093/pnasnexus/pgac228

**Published:** 2022-10-07

**Authors:** Takuya Okabe, Jin Yoshimura

**Affiliations:** Graduate School of Integrated Science and Technology, Shizuoka University, 3-5-1 Johoku, Hamamatsu 432-8561, Japan; Graduate School of Integrated Science and Technology, Shizuoka University, 3-5-1 Johoku, Hamamatsu 432-8561, Japan; Department of International Health, Institute of Tropical Medicine, Nagasaki University, 1-14 Bunkyōmachi, Nagasaki 852-8521, Japan; Department of Environmental and Forest Biology, State University of New York College of Environmental Science and Forestry, Syracuse, NY 13210, USA; Marine Biosystems Research Center, Chiba University, Uchiura, Kamogawa, Chiba 299-5502, Japan; University Museum, University of Tokyo, Bunkyo-ku, Tokyo 113-8654, Japan; Department of Biological Science, Tokyo Metropolitan University, Hachioji, Tokyo 192-0397, Japan

**Keywords:** risk-spreading, diversification, variance reduction, geometric-mean fitness, mean-variance trade-off

## Abstract

The trade-off between short-term success and long-term sustainability is a common subject of great importance both in the biological evolution of organisms and in the economic activities of human beings. In evolutionary biology, bet-hedging theories have described it as the trade-off between the (within-generation) arithmetic mean fitness and the (between-generation) geometric mean fitness of a genotype. Accordingly, bet-hedging strategies observed in various organisms are regarded as optimizing the geometric mean fitness. To increase the geometric mean fitness signifies to suppress the between-generation variance in the mean fitness. Thus, this view is consistent with mean-variance portfolio analysis in which the standard deviation of a portfolio is regarded as a measure of risk. In the present study, we provide yet another measure of long-term sustainability, which is based on minimization of the probability of extinction/bankruptcy that randomly varying population/asset size after a long time becomes less than a certain small value. We present results for representative examples to show that the present criterion gives a qualitatively similar but quantitatively different prediction from the traditional ones. In particular, we emphasize that maximizing survival probability (i.e. minimizing extinction probability) is equivalent neither to maximizing geometric mean fitness nor to minimizing variance in mean fitness, while these three are consistently related to each other.

Significance Statement Diversification by risk spreading plays a key role in sustainable development in economy and ecology. In evolutionary ecology, either geometric-mean fitness or variance in fitness has been considered a long-term measure to deal with the trade-off between short-term success and long-term sustainability. This paper presents yet another measure based on the probability of extinction after a sufficiently long time. The present criterion gives a qualitatively similar result supporting bet-hedging strategies but a quantitatively different result for optimal diversification.

## Introduction

The trade-off between short- and long-term successes is of essential importance in evolutionary ecology and sustainable economy, i.e. in the survival strategy of biological organisms and in the investment strategy of financial investors. In evolutionary biology, this trade-off between the immediate success in the next generation and the long-term survival of its lineage is of primary concern in what is called bet-hedging (1–3). Indeed, this is akin to financial hedging, where diversifying an investment portfolio protects against economic uncertainty (4). A representative example of biological bet-hedging is provided by the soil seed bank, the natural storage of plant seeds in the soil (5). In the short term, it is advantageous for the reproduction of a plant to germinate all its seeds. In the long term, however, it is advantageous to produce dormant seeds to avoid extinction by a drought. The soil seed bank is commonly seen in many plant taxa (6). Desert annual plants bet hedge by keeping a fraction of dormant seeds even when conditions are favorable (7). In mean-variance theory, the variance of asset prices is considered as a proxy for risk (4). A risk-averse investor reduces the return variance by holding a diversified portfolio. In a similar manner, biological bet-hedging is the strategy of reducing the variance in fitness, the quantitative measure of reproductive success (8). Dormant seeds play a strategic role of suppressing the variance in fitness.

However, there is an important difference between biological and economical bet-hedging. The trade-off in investment is left up to the discretion of investors. Biological systems have no agent of discretion to resolve the trade-off. In fact, biological bet-hedging is a result of the long-term success of organisms in the environment in which they evolved. To bridge between maximization of the long-term fitness and minimization of the variance, bet-hedging theory has recourse to two arguments: (a) the geometric mean fitness (5, 9) and (b) a two-allele model of population genetics (10).

The first approach puts forward the principle that the long-term fitness is the geometric mean of the growth rate per generation (11). Maximization of the geometric mean signifies minimization of the variance, owing to the mathematical property that the geometric mean, unlike the ordinary, arithmetic mean is sensitive to exceptional values of rare occurrences. In fact, it has been considered even that biological bet-hedging is defined as optimization of the (between-generation) geometric mean fitness at the cost of the (within-generation) arithmetic mean fitness (12). It is wrong to claim the geometric mean principle on the basis of the multiplicative character of the growth rate. In short, the multiplicative property leads to the multiplication of means but not to the multiplicative or geometric mean. The most convincing argument for geometric mean was provided by Lewontin and Cohen (9), who showed that, in the long term, population size should vanish with almost certainty if the geometric mean of the variable growth rates is less than unity. Thus, this argument is based not on the expected value of the random number, but on the probability of the random number to be less (or more) than a certain value. Applying this to a two-allele model of population genetics, it is concluded that the allele with the higher geometric mean fitness will go to fixation while the other allele with the lower geometric mean will go extinct almost certainly (13). Thorp made essentially the same point in regard to repeated investments (14).

The second approach, mentioned above as (b), is related but not equivalent argument specifically based on the two-allele model of population dynamics. Gillespie showed that which of two alleles has a better chance of fixation is determined by the size of $\mu _\mathit{ i} - \sigma _\mathit{ i}^2/n$, where 1 + μ_*i*_ and $\sigma _\mathit{ i}^2$ are the mean of and the variance in the offspring number of genotype *i*, respectively, and *n* is the total population size (10). Thus, lowering the variance $\sigma _\mathit{ i}^2$ increases the fixation probability, i.e. decreases the probability of extinction.

The two arguments (a) and (b) are consistently related as we see from the approximate relation (shown below in Eq. 12) (4, 9,13): *γ* ≃ *μ*exp ( − *σ*^2^/(2*μ*^2^)). For the growth rate (fitness) *l*, a random variable, *μ* = *E*(*l*) is the arithmetic mean, *σ*^2^ = *Var*(*l*) is the variance, *γ* = exp (*E*(log *l*)) is the geometric mean. Thus, the geometric mean *γ* may be increased by decreasing the variance *σ*^2^ even though the arithmetic mean *μ* is reduced. It should be noted that these results are derived based on mathematical and biological assumptions. The symbol ≃ indicates a mathematical approximation of retaining up to the second-order term in the series expansion of log *l* around the mean value *μ* of *l* (13). Fixation of either allele is concluded under the assumption that the total number *n* of the population is kept at a fixed value, even though the offspring number of each allele varies randomly (10). The latter assumption is of biological significance, for relaxing it can affect the conclusion.

In this study, therefore, we present another way of corroborating bet-hedging. In particular, we do not assume the mutual exclusion of alleles. This is important in application to nonbiological cases in which there is no counterpart of competing alleles even though bet-hedging can still remain valid similarly as in biological cases. In fact, the Kelly criterion for optimal betting strategies is based on the maximization of geometric mean growth rate (14,15). This subject has been of general interest outside the realm of biology. Accordingly, any characteristics specific to biological systems should be abstracted away while treating it on the equal footing as biological bet-hedging. Here, we argue that the long-term measure of success is provided by minimization of the probability that stochastically varying size becomes less than a certain threshold value after a sufficiently large time. This probability represents the probability of extinction in evolution and the probability of bankruptcy in real-money betting.

## Model and Results

We consider that population/asset size *S_t_* at time *t* increases with a random rate *l_t_*, so that

(1) $$\begin{equation*}
S_{\mathit{ t}+1}=l_\mathit{ t} S_{\mathit{ t}}.
\end{equation*}
$$

The growth rate *l_t_* at each time *t* is an independently and identically distributed random variable taking a positive value. The central limit theorem holds true in the limit of large *t*, where the sum in

(2) $$\begin{equation*}
\frac{1}{\mathit{ t}}\log \frac{S_\mathit{ t}}{S_0} =\frac{1}{\mathit{ t}} \sum _{\mathit{ t}=0}^{\mathit{ t}-1} \log l_\mathit{ t}
,
\end{equation*}
$$

converges to the normal distribution with mean μ_log *l*_ and variance $\sigma ^2_{\log l}/t$ with μ_log *l*_ and $\sigma ^2_{\log l}$ being the mean and variance of the random variable log *l_t_*, respectively. Accordingly, the probability that *S_t_* is less than or equal to a given value *K*, Prob(*S_t_* ≤ *K*), is evaluated by means of the cumulative distribution function (cdf) of the standard normal distribution (with mean 0 and variance1),

(3) $$\begin{equation*}
\Phi (x)=\frac{1}{\sqrt{2\pi }} \int _{-\infty }^x e^{-t^2}d\mathit{ t},
\end{equation*}
$$

such that

(4) \begin{eqnarray*} {\rm Prob}(S_\mathit{ t}\le K) &= & {\rm Prob}\left( \frac{1}{\mathit{ t}}\sum _{\mathit{ t}=0}^{\mathit{ t}-1} \log l_t \le \frac{1}{\mathit{ t}}\log \frac{K}{S_0} \right) \end{eqnarray*}

(5) \begin{eqnarray*} &=&\Phi \left( \frac{\frac{1}{\mathit{ t}} \log \frac{K}{S_0}-\mu _{\log l}}{\sigma _{\log l}/\sqrt{\mathit{ t}}} \right). \end{eqnarray*}

Since the cdf Φ(*x*) is a monotonically increasing function of the argument *x*, minimizing the probability Prob(*S_t_* ≤ *K*) is equivalent to minimizing the argument

(6) $$\begin{equation*}
X= \frac{\frac{1}{\mathit{ t}} \log \frac{K}{S_0}-\mu _{\log l}}{\sigma _{\log l}}\sqrt{\mathit{ t}}.
\end{equation*}
$$

Therefore, we are led to the optimization problem of maximizing the ratio

(7) $$\begin{equation*}
\frac{\mu _{\log l}}{\sigma _{\log l}},
\end{equation*}
$$

owing to $X \simeq - (\mu _{\log l}/{\sigma _{\log l}}) \sqrt{t}$ for *t* ≫ log (*K*/*S*_0_)/μ_log *l*_, the ratio *K*/*S*_0_ being a fixed constant.

The present result is consistent with the previous result (9,14) that whether the population becomes extinct or grows without bound depends on the sign of μ_log *l*_, i.e. whether $e^{\mu _{\log l}}\lt 1$ or not. Accordingly, $e^{\mu _{\log l}}$ may be considered the long-term growth rate (14,16). In a discrete model, this number $e^{\mu _{\log l}}$ is nothing but the geometric mean of *l_t_*. In fact, suppose that the random variable *l_t_* takes one of discrete values *l*_1_, *l*_2_, ..., with probabilities *P*_1_, *P*_2_, ..., respectively. The probabilities *P_i_* (*i* = 1, 2, ⋅⋅⋅) add up to unity: ∑_*i*_*P_i_* = 1. The mean μ_log *l*_ and variance $\sigma ^2_{\log l}$ of log *l_t_* are given by

(8) $$\begin{equation*}
\mu _{\log l}= \sum _i \mathit{ P}_i \log l_i,
\end{equation*}
$$

and

(9) $$\begin{equation*}
\sigma ^2_{\log l}= \sum _i \mathit{ P}_i (\log l_i-\mu _{\log l})^2,
\end{equation*}
$$

respectively. Eq. 8 signifies

(10) $$\begin{equation*}
e^{\mu _{\log l}}= \displaystyle \prod _i l_i^{\mathit{ P}_i},
\end{equation*}
$$

which is the geometric mean of *l_i_*. Consequently, to maximize the geometric mean is to maximize μ_log *l*_. We stress that to maximize the long-term growth rate $e^{\mu _{\log l}}$ is not the same as to minimize the extinction probability. We are interested in making a detailed comparison of maximizing μ_log *l*_/σ_log *l*_ in Eq.7 and μ_log *l*_ in Eq. 8.

## Numerical Results

Here ,we consider two examples of risk hedging in biology and optimal hedging in repeated betting.

The first example is the risk-spreading behavior of the cabbage butterfly (*Pieris rapae*) (16,17). We consider the optimal proportion for a female butterfly to distribute her eggs into two types of habitat with different qualities. Habitat 1 is of a good quality but has a risk of death due to grazing by herbivores like cows. Accordingly, the growth rate takes either of a very small value *r*_11_ = 0.005 or a large value *r*_12_ = 5 with probabilities *P*_1_ = 1/3 or *P*_2_ = 2/3. Habitat 2 is of a bad quality but without risk, where the growth rate takes a middle value *r*_2_ = 0.7 with certainty (i.e. probability 1).

Let *f* be the ratio of the eggs deposited in variable (unpredictable) habitat 1. The rest 1 − *f* are deposited in constant (predictable) habitat 2. A fractional value of *f*, 0 < *f* < 1, signifies a risk-spreading behavior.

In the short term, the ordinary mean, or the expected value, μ = ∑_*i*_*P_i_l_i_* should be maximized, the result being optimal at *f* = 1, i.e. no risk-spreading. In the long term, however, a different result is obtained by maximizing μ_log *l*_ or μ_log *l*_/σ_log *l*_. The growth rate is a random variable taking values *l*_1_ = *fr*_11_ + (1 − *f*)*r*_2_ and *l*_2_ = *fr*_12_ + (1 − *f*)*r*_2_ with probabilities *P*_1_ = 1/3 and *P*_2_ = 2/3, respectively. Thus, μ_log *l*_ and σ_log *l*_ are evaluated from Eqs. 8 and 9.

**Fig. 1.**
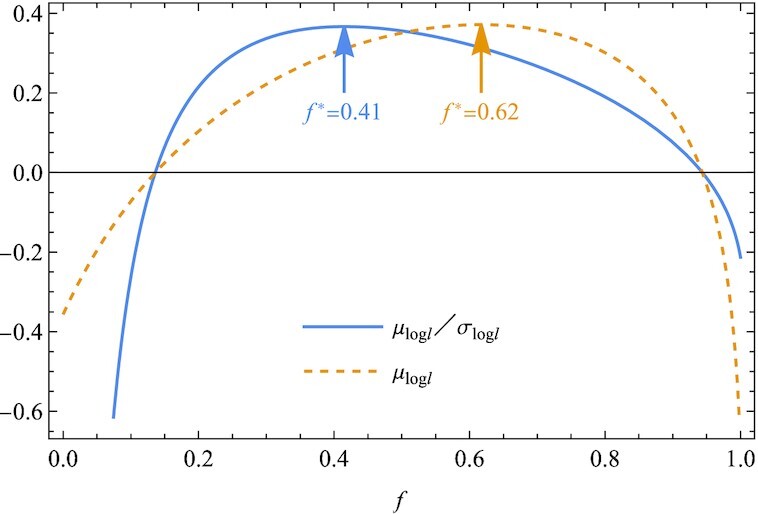
The survival probability μ_log *l*_/σ_log *l*_ and the long-term growth rate μ_log *l*_ are plotted as a function of the fraction *f* of offspring deposited in an unpredictable environment (habitat 1). An optimum at f* between 0 and 1 signifies a risk-spreading behavior. (The growth rates are *r*_11_ = 0.005 and *r*_12_ = 5 with probabilities *P*_1_ = 1/3 and *P*_2_ = 2/3 in habitat 1, while *r*_2_ = 0.7 with certainty in habitat 2).

In Fig. 1, we plot μ_log *l*_/σ_log *l*_ as well as the geometric mean μ_log *l*_ as a function of *f*. We find that both criteria, μ_log *l*_/σ_log *l*_ and μ_log *l*_, similarly predict optimality of the risk-spreading behavior, i.e. an optimal value f* between 0 and 1. Quantitatively, however, the two criteria give different values for f*. As indicated with arrows in Fig. 1, μ_log *l*_/σ_log *l*_ is maximized at f* = 0.41, while μ_log *l*_ is at f* = 0.62. The difference originates from the *f*-dependence of the variance σ_log *l*_.

**Fig. 2.**
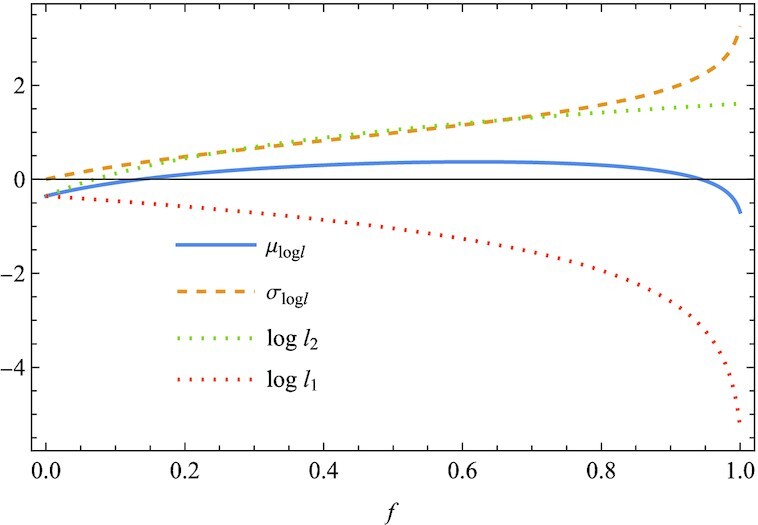
With μ_log *l*_ and σ_log *l*_ in Fig. 1, the logarithmic growth rates log *l*_1_ and log *l*_2_ under bad and good conditions, respectively, are plotted against the fraction *f*. The larger *f*, the larger the difference between log *l*_1_ and log *l*_2_, and therefore the larger the standard deviation σ_log *l*_. Thus, the optimal value f* of μ_log *l*_/σ_log *l*_ is smaller than that of μ_log *l*_ (Fig. 1) (*r*_11_ = 0.005, *r*_12_ = 5, *P*_1_ = 1/3, *P*_2_ = 2/3, and *r*_2_ = 0.7).

In Fig. 2, we plot μ_log *l*_, σ_log *l*_, log *l*_1_, and log *l*_2_ against *f*. Since μ_log *l*_ is the probability-weighted average of log *l*_1_ and log *l*_2_, all these three quantities converge toward a common value at *f* = 0, where we obtain *l*_1_ = *l*_2_ = *r*_2_ with certainty. Thus, σ_log *l*_ increases steadily from 0 to a maximum value as *f* increases from 0 to 1 (Fig. 2). Consequently, the geometric mean μ_log *l*_ overestimates the optimal value f* as compared to that of μ_log *l*_/σ_log *l*_ (Fig. 1). In other words, optimization to minimize the long-term extinction probability, based on μ_log *l*_/σ_log *l*_, predicts a more conservative result of less diversification to the high-risk, high-return investment, namely a smaller f*, than from the geometric mean μ_log *l*_. This is generally expected as it is caused because uncertainty increases the variance σ_log *l*_ (Supplementary Material). Indeed, σ_log *l*_ may be considered a measure of uncertainty. This is the key message of the present article: σ_log *l*_ plays a role.

**Fig. 3.**
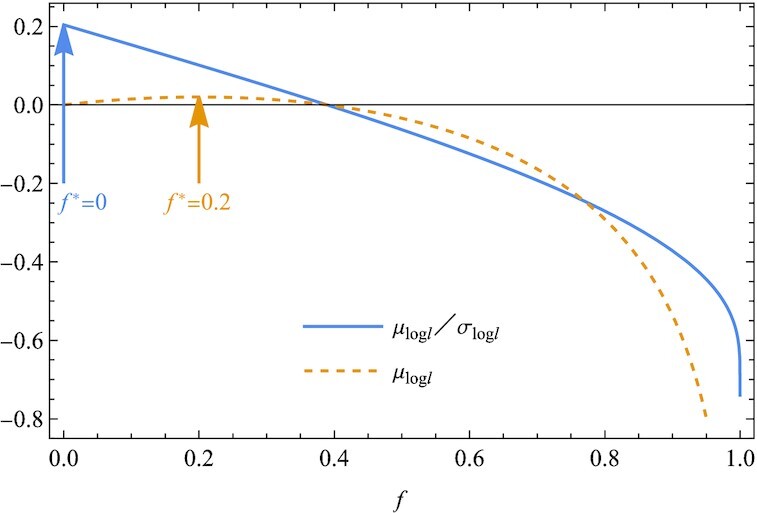
The sustainability probability μ_log *l*_/σ_log *l*_ and mean μ of the logarithmic growth rate are plotted as a function of the fraction *f* of the current wealth to wager. A positive optimum f* > 0 signifies the optimality of betting. (The growth rates are *r*_1_ = 2 and *r*_2_ = 0 on winning and losing with probabilities *P*_1_ = 0.6 and *P*_2_ = 0.4, respectively).

**Fig. 4.**
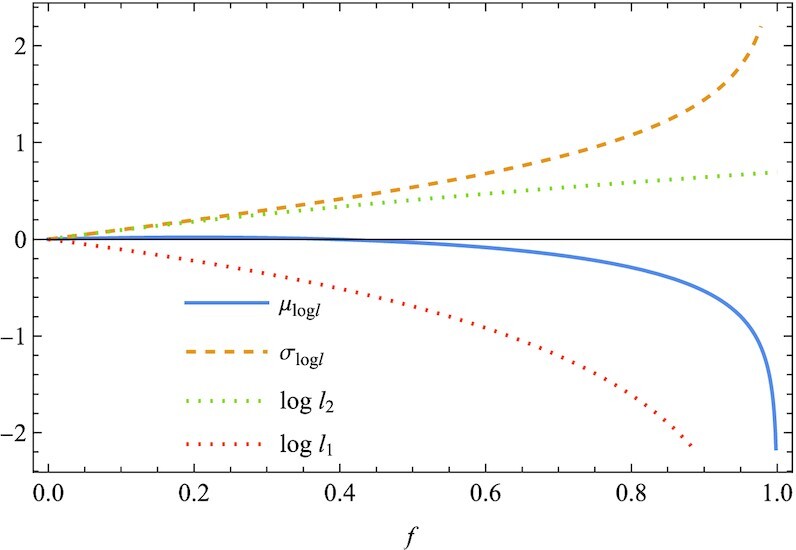
Along with μ_log *l*_ and σ_log *l*_ in Fig. 3, log *l*_1_ and log *l*_2_ on winning and losing bets, respectively, are plotted against the fraction *f*. As generally expected, σ_log *l*_ is an increasing function of *f*, so that the optimal value f* of μ_log *l*_/σ_log *l*_ is smaller than that of μ_log *l*_ (Fig. 3) (*r*_1_ = 2, *r*_2_ = 0, *P*_1_ = 0.6, and *P*_2_ = 0.4).

As a second example, we consider the optimal size for a bet, known as the Kelly criterion (14,15). In a gamble of a 60% chance of winning, the gambler receives 1-to-1 odds on a winning bet (*r*_1_ = 2), but he loses the wager (*r*_2_ = 0) with a 40% chance. The gambler repeats betting a fraction *f* of his current wealth while keeping the rest fraction 1 − *f* intact. The problem is to find optimal value of *f*. The Kelly criterion determines the optimal value f* such that the geometric mean μ_log *l*_ is maximized. Substituting *l*_1_ = *fr*_2_ + 1 − *f* with *P*_2_ = 0.4 and *l*_2_ = *fr*_1_ + 1 − *f* with *P*_1_ = 0.6, we obtain μ_log *l*_/σ_log *l*_ and μ_log *l*_, as shown in Fig. 3. Similarly as in Fig. 1, the optimal values for the two criteria μ_log *l*_/σ_log *l*_ and μ_log *l*_ are different. The present criterion μ_log *l*_/σ_log *l*_ gives f* = 0, namely no betting at all. This result is qualitatively different from what is concluded from the Kelly criterion, according to which it is optimal to bet 20% of his bankroll (f* = 0.2). As in Fig. 1, the difference originates from a monotonic change of the variance σ_log *l*_ (Fig. 4). While μ_log *l*_ has a peak at a fractional value (f* = 0.2), the peak height is too shallow to withstand a strong pressure toward *f* = 0 to minimize the denominator σ_log *l*_ in μ_log *l*_/σ_log *l*_. In fact, this is a special case. Owing to *l*_1_ = *l*_2_ = 1 at *f* = 0, μ_log *l*_/σ_log *l*_ becomes almost independent of *f*. Therefore, diversification is not favored in this case (Supplementary Material). This is a nontrivial consequence of the present study. Gambling is rational according to geometric mean (μ_log *l*_), but irrational according to the present measure (μ_log *l*_/σ_log *l*_).

## Trade-off between short and long-term benefits

The main results in the penultimate section apply to any probability distribution of *l_t_*, while we considered the simplest case of discrete choice in the last section. In this section, we consider another special case of the normal distribution in order to discuss the trade-off relation in general terms.

If the growth rate *l_i_* is normally distributed around μ, namely *l_i_* = μ + δ_*i*_, μ_log *l*_ in Eq. 8 is expanded to give

(11) \begin{eqnarray*} \mu _{\log l}&\simeq & \sum _i P_i \left( \log \mu + \frac{\delta _i}{\mu } -\frac{1}{2}\left(\frac{\delta _i}{\mu }\right)^2 \right) \nonumber \\ &=&\log \mu -\frac{\sigma ^2}{2 \mu ^2}, \end{eqnarray*}

with the variance $\sigma ^2=\sum _i P_i \delta _i^2$. Under this approximation, the geometric mean is given by

(12) $$\begin{equation*}
\gamma = e^{\mu _{\log l}} \simeq \mu \exp \left( -\frac{\sigma ^2}{2 \mu ^2} \right).
\end{equation*}
$$

Thus, the geometric mean γ may be increased by decreasing the variance σ^2^ even if the ordinary mean μ is reduced (4, 9,13). Similarly, Eq. 9 gives

(13) \begin{eqnarray*} \sigma ^2_{\log l} &=& \frac{\sigma ^2}{ \mu ^2} \left(1-\frac{\sigma ^2}{2 \mu ^2} \right), \end{eqnarray*}

so that

(14) $$\begin{equation*}
\frac{\mu _{\log l}}{\sigma _{\log l}} = \frac{ \log \mu -\frac{\sigma ^2}{2 \mu ^2} }{\sqrt{ \frac{\sigma ^2}{ \mu ^2} \left(1-\frac{\sigma ^2}{2 \mu ^2} \right) }}.
\end{equation*}
$$

This ratio is a decreasing function of *σ*^2^/μ^2^, if σ^2^/μ^2^ < 1. Consequently, maximizing μ_log *l*_/σ_log *l*_ is approximately equivalent to minimizing σ/μ. Thus, we reach a new way of justifying bet-hedging strategies of reducing σ/μ by way of the present long-term measure μ_log *l*_/σ_log *l*_.

**Fig. 5.**
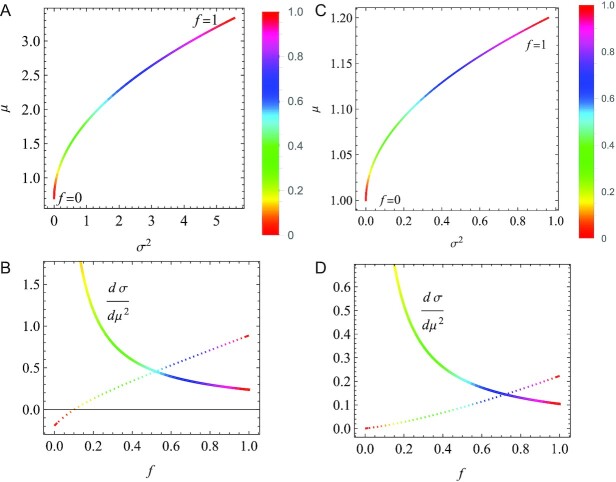
The trade-off between the mean μ and variance σ^2^ of the growth rate (A and C) and the strength of the trade-off *d*μ/*d*(σ^2^) (B and D). (A and B) The case of Figs. 1 and 2 (*r*_11_ = 0.005, *r*_12_ = 5, *P*_1_ = 1/3, *P*_2_ = 2/3, and *r*_2_ = 0.7). (C and D) The case of Figs. 3 and 4 (*r*_1_ = 2, *r*_2_ = 0, *P*_1_ = 0.6, and *P*_2_ = 0.4). Dotted curves in B and D show the right-hand side of Eq. 15.

More specifically, we need to consider that there are many cases in which mean μ and variance σ^2^ do not vary independently from each other. It is generally expected a trade-off relation between μ and σ^2^, to the effect that the larger μ, the larger σ^2^. Indeed, the two examples in the last section exemplify this trade-off relation. In panels A and C of Fig. 5, we show the parametric plots of μ versus σ^2^ as implicit functions of *f*.

In investment decisions, the trade-off is resolved by the individual investor. In evolutionary ecology, the trade-off is resolved by natural selection. In fact, many organisms appear to prioritize the long-term survival (by reducing the variance σ^2^) over the short-term success (of increasing the mean μ) (18,19). The trade-off relation between μ and σ^2^ suggests that the derivative *d*μ/*d*(σ^2^) serves as the strength of the trade-off, in terms of which the condition for bet-hedging may be stated theoretically. The condition for the chances of survival to increase as σ^2^ decreases is given by ∂(μ_log *l*_/σ_log *l*_)/∂(σ^2^) < 0, from which we obtain

(15) $$\begin{equation*}
\frac{d \mu }{d (\sigma ^2)} \lt \frac{\mu (\sigma ^2 + 2(\mu ^2-\sigma ^2)\log \mu )}{4 (\mu ^2+(\mu ^2-\sigma ^2)\log \mu )}
\end{equation*}
$$

by differentiating Eq. 14. This inequality represents that the trade-off (*dμ*/*d*(*σ*^2^)) should be weaker than a threshold value on the right-hand side.

Assuming that the statistical properties of interest are satisfactorily represented with the mean *μ* and variance *σ*^2^, we may apply the above result to the two examples in the last section. In panels Fig. 5B and D, the derivative *d*μ/*d*(σ^2^) is plotted with a solid curve, along with the right-hand side of Eq. 15 with a dotted curve. For the two examples, the inequality in Eq. 15 is met for *f* > 0.52 (Fig. 5B) and *f* > 0.72 (Fig. 5D). Thus, we corroborate the advantage of the long-term benefit over the short-term benefit (*f* = 1).

## Discussions

To maximize geometric mean, or μ_log *l*_ in Eq. 8, is analogous to maximizing the logarithmic utility in the expected utility theory, where a concave utility function like the logarithim indicates risk aversion. Similarly to the logarithmic utility, μ_log *l*_ is shown to be a concave function of *f*, whereas the concavity alone does not rationalize bet-hedging (0 < *f* < 1). Bet-hedging is concluded only if μ_log *l*_ is unimodal (Fig. 1). Nonetheless, it is instructive to draw a parallel between μ_log *l*_ and the logarithmic utility. The present measure μ_log *l*_/σ_log *l*_ falls outside the scope of the expected utility hypothesis, as it is not represented as the expectation of a utility function. However, it is worth noting that this fractional form has been investigated as a promising generalization of the expected utility theory to cope with the Allais paradox (20–22). We underline that the geometric mean is not essential to bet-hedging, as the logarithmic utility is less than necessary for risk aversion.

In this study, we focused on the probability in Eq. 4. This is because the model in Eq. 1 does not lead to extinction in the true sense of the word, owing to the assumption that *l_t_* is positive. This assumption is indispensable in order not to make the logarithm log *l_t_* ill-defined. If we relax this assumption by allowing the catastrophic event of *l_t_* = 0 with probability *P*_0_, the long-term extinction, *S_t_* = 0 as *t* → ∞, is concluded with absolute certainty, in so far as *P*_0_ is not identically zero for all *t*. In real life, the size *S_t_* of an ecological population takes an integer value, as it consists of individuals. Extinction occurs when *S_t_* hits the bottom value zero. We may equate the probability of this event to Eq. 4 with *K* of about or less than one. In the economical context, whether a definable minimum exists is generally not so clear-cut, but it is not unreasonable to assume a minimum *K* that, when fallen below, permits no return. This is the rationale behind the present criterion. Essentially the same analysis may be made by lifting the assumption of *l_t_* being independent and identically distributed, while the probability of extinction becomes a product of nested conditional expectations as treated in dynamic pro-gramming (23).

It should be remarked that the idea of measuring extinction in terms of the mean and variance of the probability distribution is not new in itself. Indeed, the ratio of the deviation to the mean squared (i.e. σ^2^/μ^2^) has been considered as an extinction measure in recent studies on the persistence of species populations with spatial heterogeneity (24, 25). The rationale is that the probability of extinction should increase as this ratio increases under the plausible assumption that the higher order moments than these first two, mean and variance, do not play an important role to the statistical properties of interest. While this argument appears generally valid, it is not specific enough to distinguish μ_log *l*_/σ_log *l*_ and μ/σ in the previous sections. Optimization of these two is not exactly equivalent to each other, as shown by Eq. 14. The present study indicates that the former is proper. The mean and variance should be those of the logarithmic growth rate instead of the growth rate itself. As a matter of fact, this specific result is due to our model assumption that the growth rate *l_t_* varies independently of size *S_t_*, which enables us a facile analysis in terms of a stationary distribution of *l_t_*. This assumption appears valid at least as a first approximation in the economic context, for, in general, asset size barely influences a fund’s performance. However, relaxing this assumption provides insight into the extinction problem in the evolutionary ecological context. Spatial heterogeneity in the population has been considered a key factor for the persistence observed in nature (26, 27). In a heterogeneous environment, the persistence of a population may be promoted through a “density”-dependent birth/death rate, i.e. the *S_t_*-dependent *l_t_* (25).

In applying the present results to empirical studies on biological bet-hedging, we need to be careful about how they relate to commonly used terminology. We may regard *l_t_* as the fitness at generation *t* of individuals with a focal genotype. As a random variable, *l_t_* takes one of alternative values, depending on different environmental conditions. The mean μ_log *l*_ and variance σ_log *l*_ are identified with the “between-generation” mean and variance, respectively, of the logarithmic fitness, if we consider a sufficiently large number of generations to experience all the possible conditions. A particular value taken by the random variable *l_t_* is arithmetic mean fitness (AFT) (12), and arithmetic mean of AMFs (AMAMF) (12) corresponds to the “between-generation” mean of the fitness, namely μ. Geometric mean fitness (GMF) is μ_log *l*_ exponentiated, namely γ. We do not delve into a conundrum of what should and should not be called bet-hedging, i.e. whether it requires a trade-off or not (1–3,12,28,29). We put forward that the ratio μ_log *l*_/σ_log *l*_ provides the long-term measure to rationalize bet-hedging by means of diversified strategies.

In closing, we point out a novel insight gained from the present result. It is known that bet-hedging is not favored in fine-grained environments (28, 30). Individuals in fine-grained environments experience different environments in every generation. According to the law of large numbers, the range of variation of *l_t_* diminishes rapidly as population size increases. In short, stochasticity has no sizable effect on within-generation bet-hedging. If we view this nullification of stochastic uncertainty as a strategic adaptation, it is most adequately explained as a result of the decrease in the variance σ_log *l*_, without any resort to the mean μ_log *l*_. In fact, there is no point in having recourse to the latter, or geometric mean, as it is no different from arithmetic mean μ in this case.

## Conclusion

In conclusion, we presented a new principle of diversification strategies, or bet-hedging, which is based on the maximization of the survival probability in the long term. The principle is to maximize the ratio of mean and standard deviation of the logarithmic growth rate. As compared with a conventional method by geometric mean, the present criterion gives a qualitatively similar but quantatively distinct result on the optimal fraction for diversification. Lastly, we should note that empirical data on bet-hedging strategies are not comprehensive enough to distinguish which method of bet-hedging theory is better than another (12, 19), while some empirical studies have shown evidence in qualitative agreement with the optimization of geometric mean fitness (7, 31). A more quantitative analysis of our results will have to wait for a future study.

## Acknowledgments

The authors thank the anonymous reviewers for their careful reading of our manuscript and their many insightful comments and suggestions.

## Supplementary Material

pgac228_Supplemental_Files

## Data Availability

This article has no additional data.
