# Supplementary material for: A new long-term measure of sustainable growth under uncertainty
Source: PNAS Nexus. 2022 Oct 7;1(5):pgac228. doi: 10.1093/pnasnexus/pgac228 (PMC9802095; doi:10.1093/pnasnexus/pgac228)
Supplement: pgac228_Supplemental_Files [file pgac228_supplemental_files.zip › PNASNEXUS-PNASNEXUS-2022-00658-s03.pdf]

Supplementary Material for

A new long-term measure of sustainable growth under uncertainty

Takuya Okabe and Jin Yoshimura

Supplementary Text

We are often interested in a choice between two fates, with growth rates l_1 and l_2 which deviate from each other as a (diversification) parameter f increases. Accordingly, let us assume that they vary as

$$l_1 = l_0 - \delta_1 f, \tag{1}$$

and

$$l_2 = l_0 + \delta_2 f, \tag{2}$$

where δ_1 and δ_2 are assumed positive.

A straightforward calculation gives

$$\mu_{\log l} \simeq \log l_0 - \frac{p_1\delta_1 - p_2\delta_2}{l_0} f \tag{3}$$

and

$$\sigma_{\log l} \simeq \frac{\sqrt{p_1 p_2}(\delta_1 + \delta_2)}{l_0} f, \tag{4}$$

to the linear order of f. While δ_1 and δ_2 need not be both positive, it should be noted that $\sigma_{\log l}$, the square root of $\sigma^2_{\log l}$, must be a positive number. Three cases are distinguished according to the value of l_0.

Case 1: $l_0 < 1$. This is the case of the first example in the main text (i.e., $l_0 = r_2 = 0.7$, $\delta_1 = r_2 - r_{11} = 0.7 - 0.005$ and $\delta_2 = r_{12} - r_2 = 5 - 0.7$). In this case, the increase in f increases $\mu_{\log l}/\sigma_{\log l}$ owing to $\log l_0 < 0$. Therefore, diversification is favored by $\mu_{\log l}/\sigma_{\log l}$. The assumed values of the parameters give $p_1\delta_1 - p_2\delta_2 < 0$, so that $\mu_{\log l}$ increases as f does. In other words, the geometric mean principle yields a qualitatively similar result. However, it is important to remark that these two criteria are not equivalent to each other. In principle, there can be cases where diversification is favored despite the decrease in the geometric mean $\mu_{\log l}$.

Case 2: $l_0 = 1$. This is the case of the second example in the main text ($\delta_1 = 1 - r_2 = 1$ and $\delta_2 = r_1 - 1 = 1$). In this case, owing to $\log l_0 = 0$, $\mu_{\log l}/\sigma_{\log l}$ becomes independent of f, i.e., diversification is not favored against no diversification $f = 0$. Consequently, $\mu_{\log l}/\sigma_{\log l}$ and $\mu_{\log l}$ lead to different conclusions.

Case 3: $l_0 > 1$. Owing to $\log l_0 > 0$, $\mu_{\log l}/\sigma_{\log l}$ decreases as f increases, i.e., diversification is disfavored, at least for a small value of f. This is a trivial case of little interest, in that population/asset size never goes extinct as it grows without bound.
